# The Dental Meetings at Old Point, Va

**Published:** 1894-08

**Authors:** 


### Editorial, Etc.

THE DENTAL MEETINGS AT OLD POINT, VA.—The Dental meetings held during the present month, August, at Old Point, Virginia, were unusually pleasant and interesting. The gathering of Dentists from Canada to Texas in attendance on the meetings of the "Virginia Dental," the "National Association of Dental Faculties," the "National Association of Boards of Dental Examiners," and the "Southern" and "American Dental Associations," rendered the occasion one of peculiar interest, and brought together practitioners who had been separated, in a number of cases, for many years. The North, South, East and West joined in contributing to the pleasure of association for professional interests, and each meeting was in the highest degree a success. The weather was very pleasant, the temperature agreeable, and the well known Hygeia Hotel maintained its reputation as a seaside resort of the first class.

Two United States Training Vessels anchored a few hundred yards from the beach in the world renowned Hampton Roads, and filled with cadets from Annapolis, added to the gayety of the scene and the pleasure of the guests especially the young lady portion.

The display of dental goods by the many Dental Depots represented at these meetings, was exceptionally fine and interesting. The villiage of Hampton, the oldest Church in Virginia, the Soldiers Home, and the Hampton Indian School proved attractive for Sunday visits; also Norfolk, Portsmouth and Newport News, and the scene of the great Monitor Naval engagement, all but a short sail, and the two latter in sight, from the Point.

The old Fortress Monroe, now used as an Artillery training school was also a point of interest to the many visitors. Fishing and salt water bathing were also attractive to many; although indulgence in oysters and soft crabs to the unacclimated, especially the latter when accompanied with milk proved too much of a luxury to many who could not resist such a temptation during the dog days. But two of the days passed at Old Point proved warm, and only to those who were unacquainted with the cool spots always to be found at the Hygeia by the initiated. The business transacted from the 2nd to the 10th of August by the different Associations was interesting and instructive, and the sail to the Capes on an invitation tendered to all the delegates by the Virginia State Dental Association, was delightful and heartily enjoyed by all who participated in it. The seashore will again welcome the American next year, while the Southern will assemble among the mountains of the South.

